# Author Correction: The medial septum controls hippocampal supra-theta oscillations

**DOI:** 10.1038/s41467-023-43190-6

**Published:** 2023-11-21

**Authors:** Bálint Király, Andor Domonkos, Márta Jelitai, Vítor Lopes-dos-Santos, Sergio Martínez-Bellver, Barnabás Kocsis, Dániel Schlingloff, Abhilasha Joshi, Minas Salib, Richárd Fiáth, Péter Barthó, István Ulbert, Tamás F. Freund, Tim J. Viney, David Dupret, Viktor Varga, Balázs Hangya

**Affiliations:** 1 Lendület Laboratory of Systems Neuroscience, Institute of Experimental Medicine, Budapest, Hungary; 2 Department of Biological Physics, Institute of Physics, Eötvös Loránd University, Budapest, Hungary; 3 Subcortical Modulation Research Group, Institute of Experimental Medicine, Budapest, Hungary; 4 Medical Research Council Brain Network Dynamics Unit, Nuffield Department of Clinical Neurosciences, University of Oxford, Oxford, UK; 5 Department of Anatomy and Human Embryology, Faculty of Medicine and Odontology, University of Valencia, Valencia, Spain; 6 Faculty of Information Technology and Bionics, Pázmány Péter Catholic University, Budapest, Hungary; 7 Department of Pharmacology, University of Oxford, Oxford, UK; 8 Institute of Cognitive Neuroscience and Psychology, Research Centre for Natural Sciences, Budapest, Hungary; 9 Laboratory of Cerebral Cortex Research, Institute of Experimental Medicine, Budapest, Hungary

**Keywords:** Neuroscience, Neural circuits

Correction to: *Nature Communications* 10.1038/s41467-023-41746-0, published online 10 October 2023

The original version of this Article contained an error in the second sentence of the Acknowledgements section, which should have read:

“We thank István Katona for kindly providing access to an in vitro electrophysiology setup and Albert M. Barth for additional electrophysiology equipment.”

In addition, the grant number NKFIH FK 129019 for Márta Jelitai and Viktor Varga was omitted.

The original article has been corrected.

