# Supplementary material for: The medial septum controls hippocampal supra-theta oscillations
Source: Nat Commun. 2023 Oct 10;14:6159. doi: 10.1038/s41467-023-41746-0 (PMC10564782; doi:10.1038/s41467-023-41746-0)
Supplement: Supplementary file 1 — Supplementary Information [file 41467_2023_41746_MOESM1_ESM.pdf]

The medial septum controls hippocampal supra-theta oscillations

Király et al.

Supplementary Information

Supplementary Figures

Supplementary Figure 1

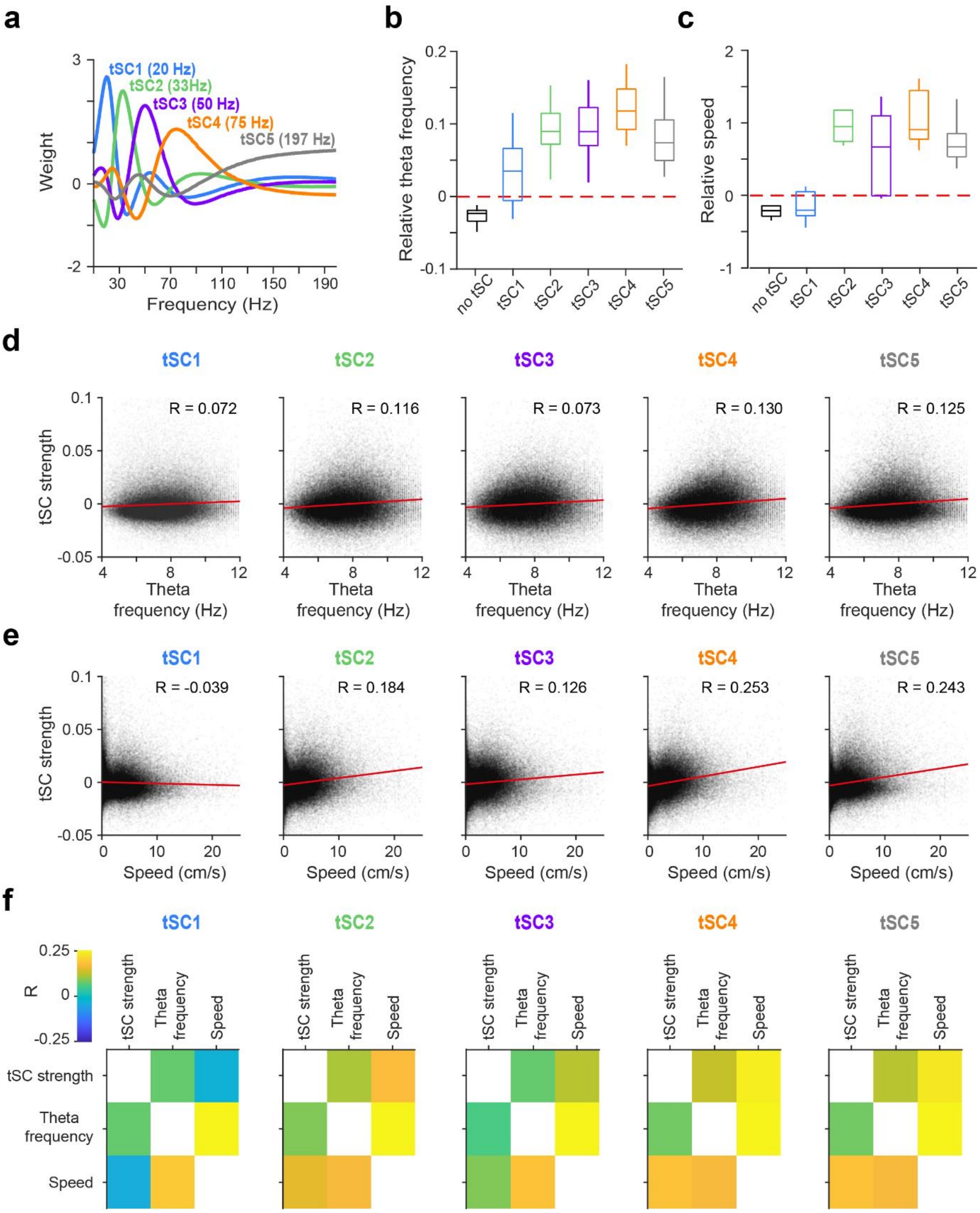

Supplementary Figure 1. Expression of tSCs depends on theta frequency and locomotion speed in freely moving mice.

a The frequency content of each tSC in the example session in Figure 1f. Peak frequencies are shown in brackets. **b,c** Box-whisker plots showing the frequency of theta cycles (**b**, n = 31 sessions**)** and the speed of the animals (**c**, n = 29 sessions) during theta cycles expressing different tSCs, relative to the average over all cycles (red dashed lines). Boxes and whiskers show median, interquartile range and non-outlier range. **d,e** tSC strength as a function of theta frequency (**d**) and the speed of the animal (**e**) during theta cycles expressing different tSCs. Red lines show linear regression model fits; R values represent Spearman correlation coefficient. **f** Partial correlations between tSC strength, theta frequency and animal speed for theta cycles strongly expressing a given tSC. Upper right triangles of the correlation matrices show correlation between the corresponding two variables without controlling for the third variable (see panels **d** and **e**), while the lower left triangles represent partial correlations with controlling for the effect of the third variable. All correlations are significant (Spearman's correlation, two-sided tests with Bonferroni's correction for multiple comparisons, $p < 0.001$). Source data are provided as a Source Data file.

Supplementary Figure 2

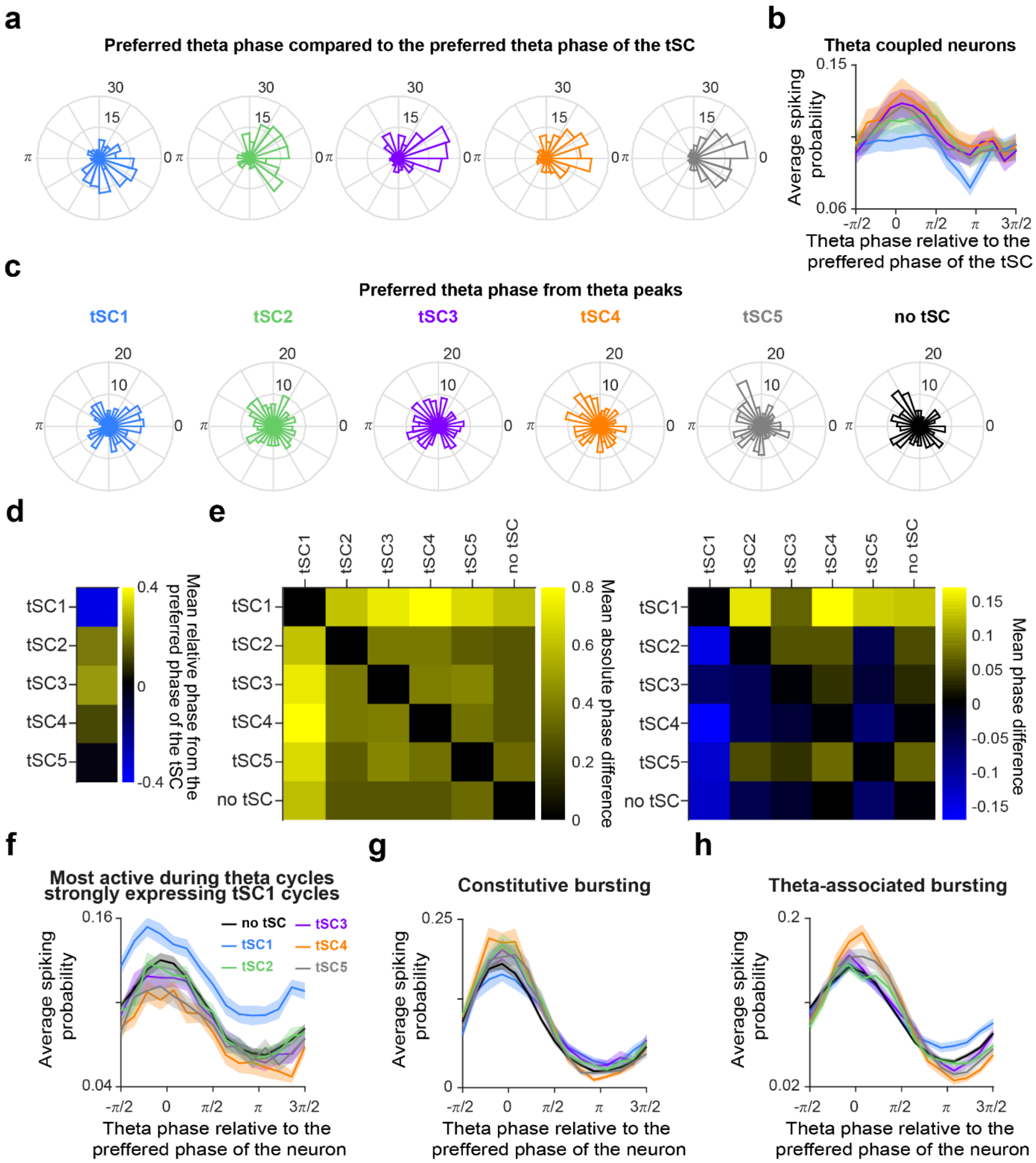

Supplementary Figure 2. MS neurons' phase coupling to hippocampal theta depends on tSCs

a Histograms of preferred phase of the theta-coupled neurons relative to the preferred phase of the concurrent tSCs. **b** Average theta phase histogram (relative to the preferred phase of the different tSCs) of theta-coupled MS neurons during cycles expressing the given tSC. **c** Histogram of the preferred theta phase of the theta-coupled neurons during theta cycles expressing different tSCs. **d** Mean relative preferred theta phase of theta-coupled MS neurons in theta cycles expressing different tSCs. **e** Mean absolute (left) and signed (right) preferred theta phase difference between theta cycles expressing different tSCs. **f-h** Average theta phase histogram (relative to the preferred phase) of MS neurons most active during tSC1 cycles (**f)**, constitutive bursting (**g)** and theta-associated bursting MS neurons (**h**) during theta cycles expressing different tSCs. Error shades show the standard error of the mean. Source data are provided as a Source Data file.

Supplementary Figure 3

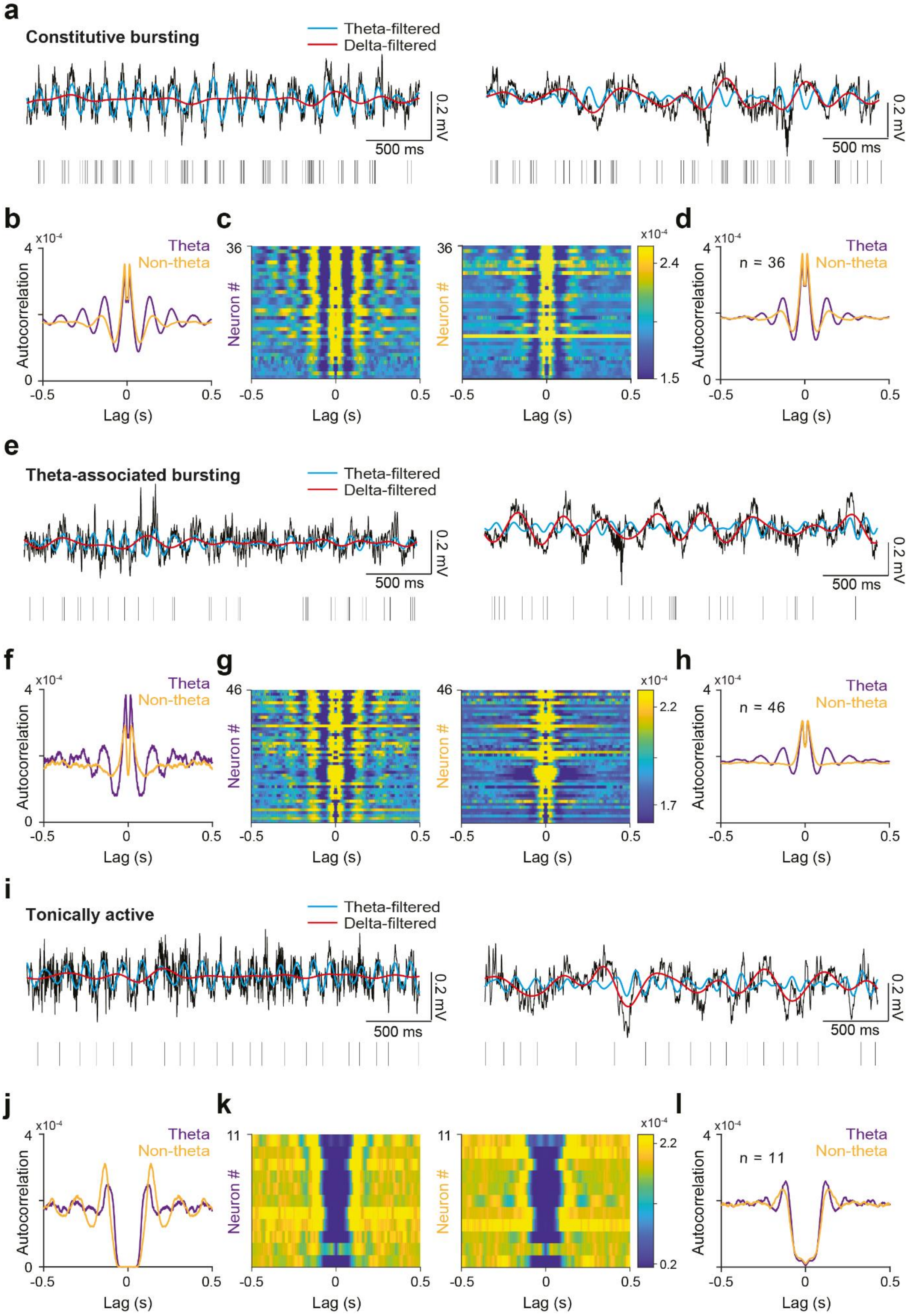

Supplementary Figure 3. Categorizing MS neurons based on their rhythmic firing properties.

a Hippocampal CA1 LFP (top; black, raw; blue, theta band filtered; red, delta band filtered) with spike raster of a constitutive bursting cell during theta (left) and non-theta segments (right). **b** Autocorrelogram of the example neuron in panel **a** during theta (purple) and non-theta (orange) segments. **c** Autocorrelograms of all constitutive bursting MS neurons during theta (left) and non-theta segments (right). **d** Average autocorrelogram of constitutive bursting MS neurons during theta (purple) and non-theta (orange) segments. Constitutive bursting neurons showed theta-rhythmic bursting activity during both theta and non-theta segments and a strong phase locking to CA1 theta. **e** Hippocampal CA1 LFP (top; black, raw; blue, theta band filtered; red, delta band filtered) with spike raster of a theta-associated bursting MS neuron during theta (left) and non-theta segments (right). **f** Autocorrelogram of the example neuron in panel **e** during theta (purple) and non-theta (orange) segments. **g** Autocorrelograms of all theta-associated bursting MS neurons during theta (left) and non-theta segments (right). **h** Average autocorrelogram of theta-associated bursting MS neurons during theta (purple) and non-theta (orange) segments. Theta-associated bursting neurons fired theta-rhythmic bursts of action potentials during CA1 theta but were not rhythmic during non-theta segments. They exhibited significant phase coupling to CA1 theta. **i** Hippocampal CA1 LFP (top; black, raw; blue, theta band filtered; red, delta band filtered) with spike raster of a tonically active MS neurons during theta (left) and non-theta segments (right). **j** Autocorrelogram of the example neuron in panel **i** during theta (purple) and non-theta (orange) segments. **k** Autocorrelograms of all tonically active MS neurons during theta (left) and non-theta segments (right). **l** Average autocorrelogram of tonically active MS neurons. Tonically active cells fired regularly at theta-band frequencies irrespective of CA1 theta oscillations (rhythmicity frequency based on autocorrelation peak, median ± standard error of the median, 8.40 ± 0.37 Hz during theta, 7.15 ± 0.4 Hz during non-theta segments), but showed little to no phase locking to hippocampal theta[52]. Source data are provided as a Source Data file.

Supplementary Figure 4

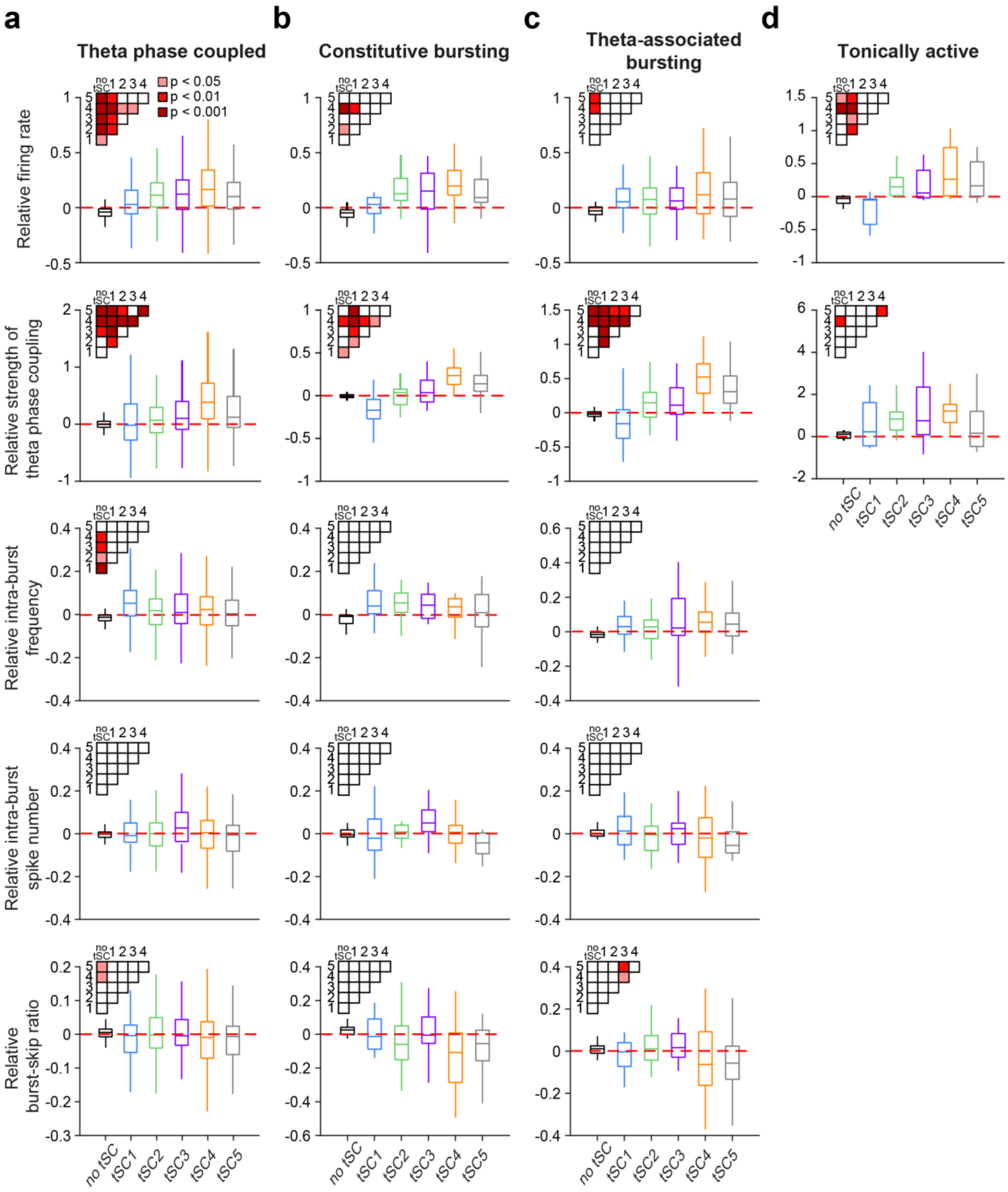

Supplementary Figure 4. Firing parameters of MS neuron populations in theta cycles expressing different tSCs

a Firing rate, phase coupling strength, intra-burst frequency, mean intra-burst spike number and burst-skip ratio distributions of the theta-coupled MS neurons (n = 181**)**, in the function of tSC presence. **b** The same for constitutive bursting MS neurons (n = 19). **c** The same for theta-associated bursting MS neurons (n = 34). **d** The same for tonically active MS neurons (n = 10). All parameters are expressed relative to the average over all cycles (red dashed line). Boxes and whiskers show median, interquartile range and non-outlier range. Differences were statistically tested with two-sided repeated measures ANOVA, followed by Tukey's test for post hoc comparisons. Significant differences are indicated by the color-coded matrices in the insets. Source data are provided as a Source Data file.

Supplementary Figure 5

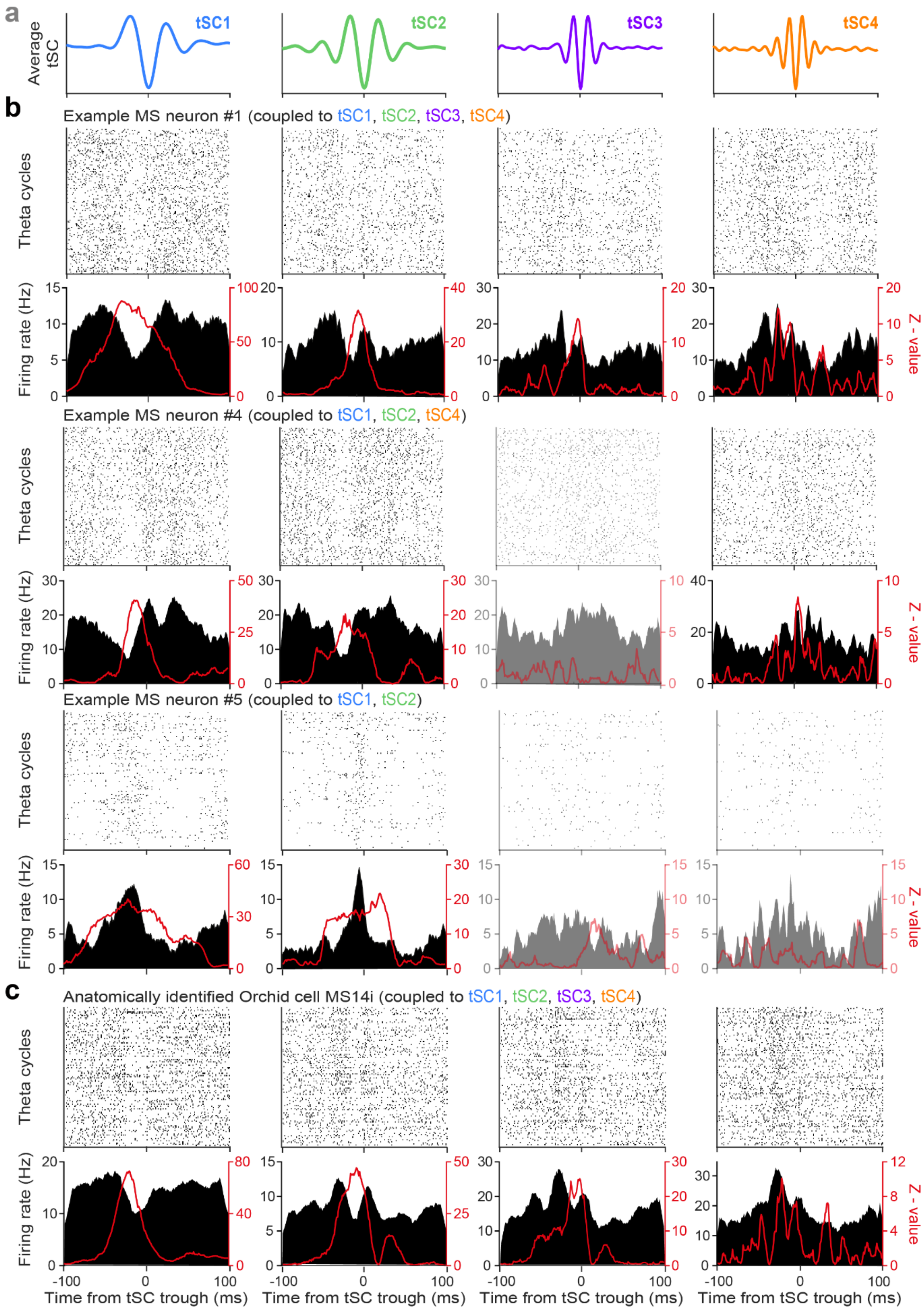

Supplementary Figure 5. Examples of tSC-coupled MS neurons

a Average tSC signals from an example session. **b** Firing pattern of three extracellularly recorded example MS neurons (example neuron #1 is the same as in Figure 3a). Top, spike raster aligned to the most negative tSC troughs within each theta cycle. Bottom, peri-event time histograms (PETHs) corresponding to each spike raster (black; y-axis on the left) and Rayleigh's Z-value as a function of temporal offset between hippocampal tSCs and MS spike trains (red; y-axis on the right). **c** Same as panel **b** for a juxtacellularly labeled example Orchid neuron (MS14i).

Supplementary Figure 6

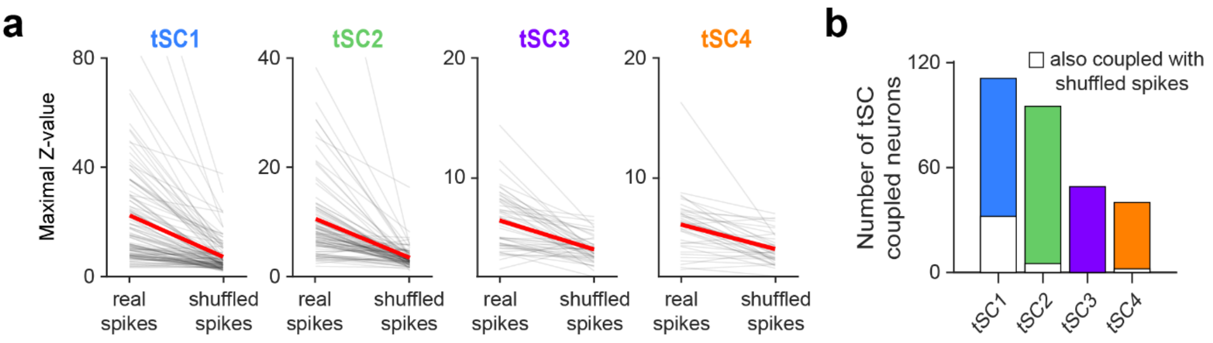

Supplementary Figure 6. Shuffling control for tSC coupling

a The maximal value of Rayleigh's Z statistic for neurons coupled to each tSC compared to the maximal Z-value computed with the spikes shuffled across theta cycles. **b** Number of neurons phase-coupled to each tSC. White bars indicate the number of neurons still significantly coupled after spikes were shuffled across theta cycles. Source data are provided as a Source Data file.

Supplementary Figure 7

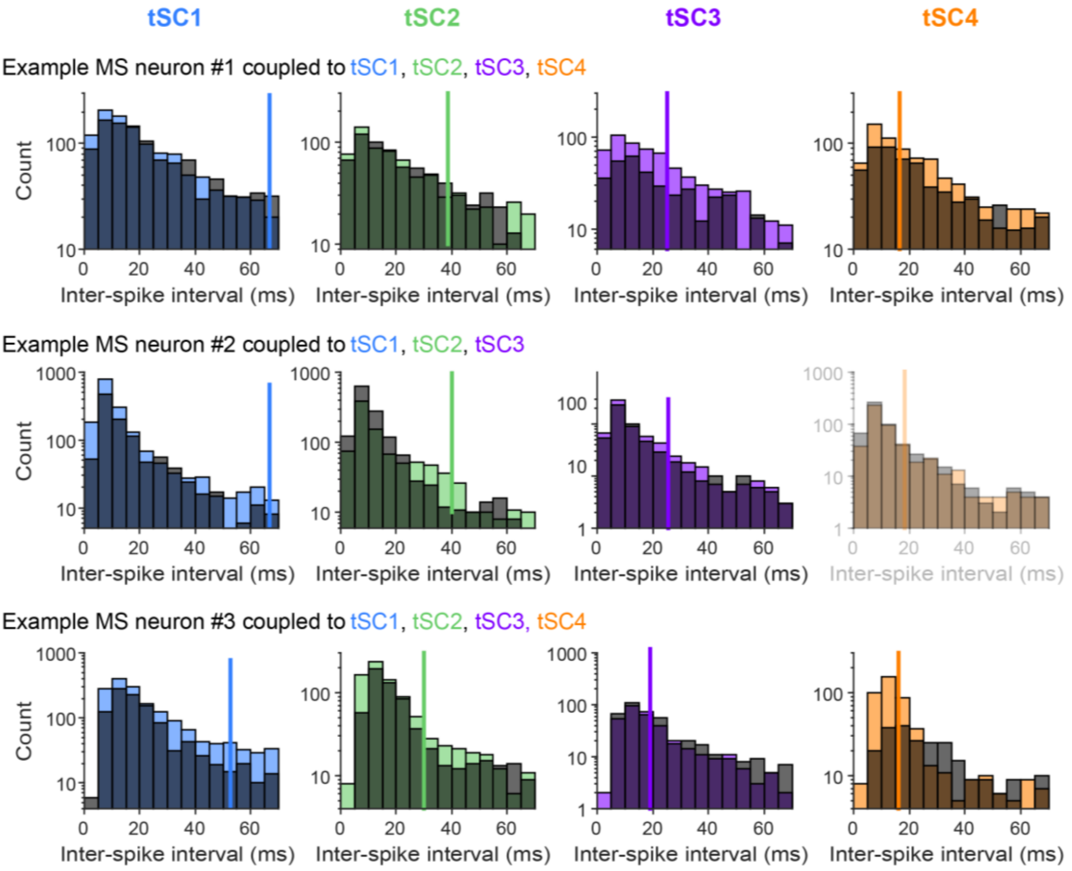

Supplementary Figure 7. Intra-burst frequency of MS neurons is correlated with CA1 tSC presence.

Inter-spike interval histograms of three extracellularly recorded example MS neurons coupled to multiple tSCs (same examples as in Figure 3) during theta cycles strongly expressing a given tSC (colored bars) compared to theta cycles expressing the given tSC the least (gray bars). The tSC4 panel of the example neuron #2 is faded to indicate that this neuron was not coupled to tSC4. The colored vertical lines indicate the frequency of the corresponding tSC. Note the ISI surplus around the corresponding frequency of the concurrent tSC if the neuron was coupled to that tSC in example MS neuron #2 and #3; see also example MS neuron #2 during tSC4 cycles, where no significant coupling was detected and no ISI surplus around the frequency of tSC4 was found, accordingly.

Supplementary Figure 8

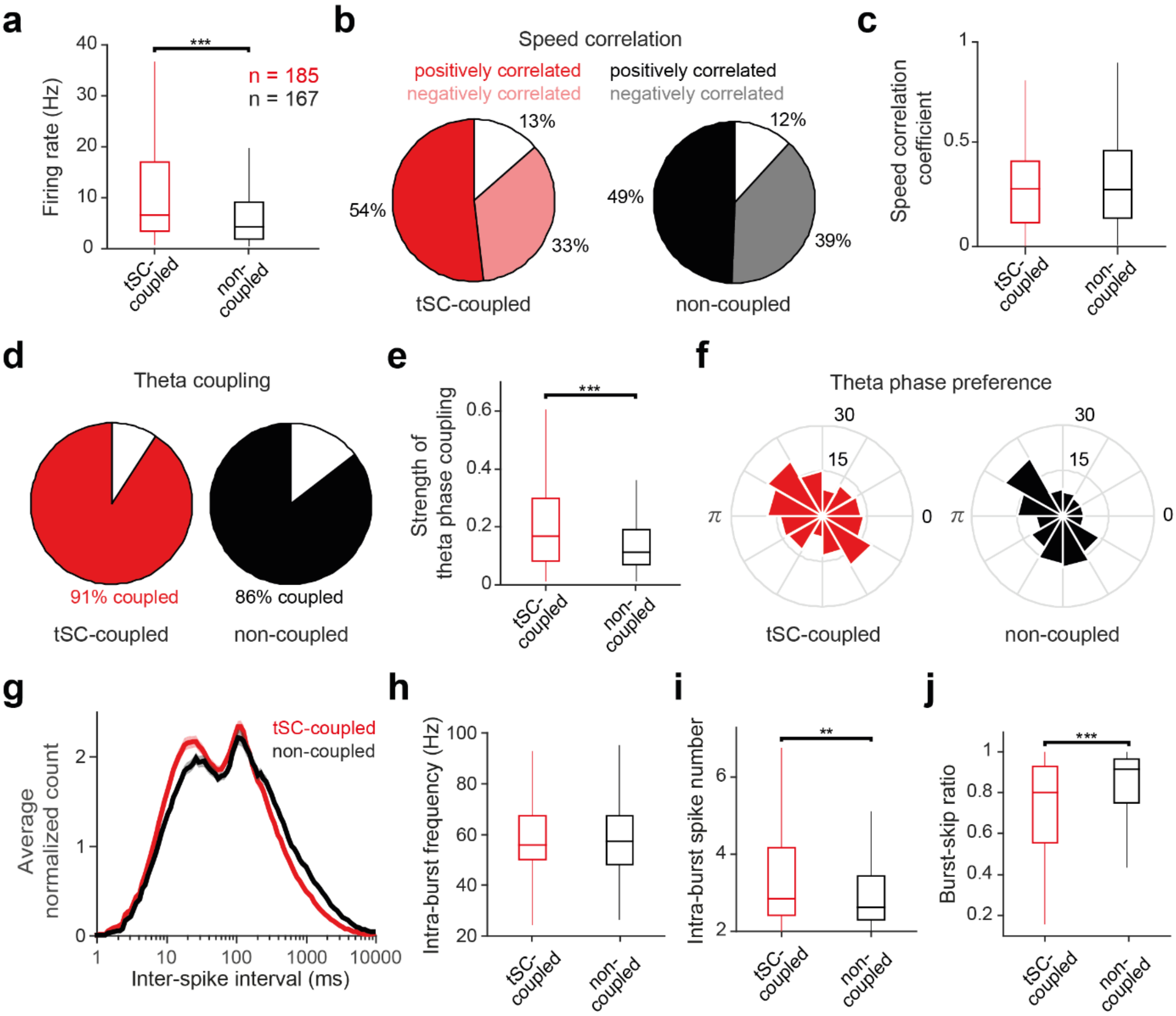

Supplementary Figure 8. Firing parameters of tSC-coupled and not coupled MS neurons

a Mean firing rate distribution of tSC-coupled (n = 185) and non-coupled (n = 167) neurons ($p = 1 \times 10^{-5}$, two-sided Mann-Whitney U-test). **b** Proportion of MS neurons with significant speed correlation (Spearman correlation, two-sided test, $p < 0.01$) in the two MS neuron groups (chi-squared test, p = 0.555). **c** Spearman correlation coefficient between the firing rate and the speed of the animal (n = 171 coupled and n = 156 non-coupled neurons, p = 0.699, two-sided Mann-Whitney U-test). **d** Proportion of theta-coupled (two-sided Rayleigh test, $p < 0.01$) neurons (chi-squared test, p = 0.130). **e** Theta-coupling strength distribution measured by the mean resultant length. (n = 185 coupled and n = 167 non-coupled neurons, $p = 3 \times 10^{-4}$, two-sided Mann-Whitney U-test). **f** Phase histogram of the preferred theta phase of the two neuron groups. **g** Average inter-spike interval histogram of the two neuron populations. The error shades show the standard error of the mean. **h-j** Distribution of burst parameters in the two MS neuron groups (n = 185 coupled and n = 167 non-coupled neurons). **h**, Intra-burst

frequency ($p = 0.581$, two-sided Mann-Whitney U-test). **i**, Intra-burst spike number ($p = 0.0099$, two-sided Mann-Whitney U-test). **j**, proportion of skipped theta cycles, termed ‘burst-skip ratio’ ($p = 5 \times 10^{-6}$, two-sided Mann-Whitney U-test). Boxes and whiskers show median, interquartile range and non-outlier range. *, $p < 0.05$; **, $p < 0.01$; ***, $p < 0.001$; Mann-Whitney U-test. Source data are provided as a Source Data file.

Supplementary Figure 9

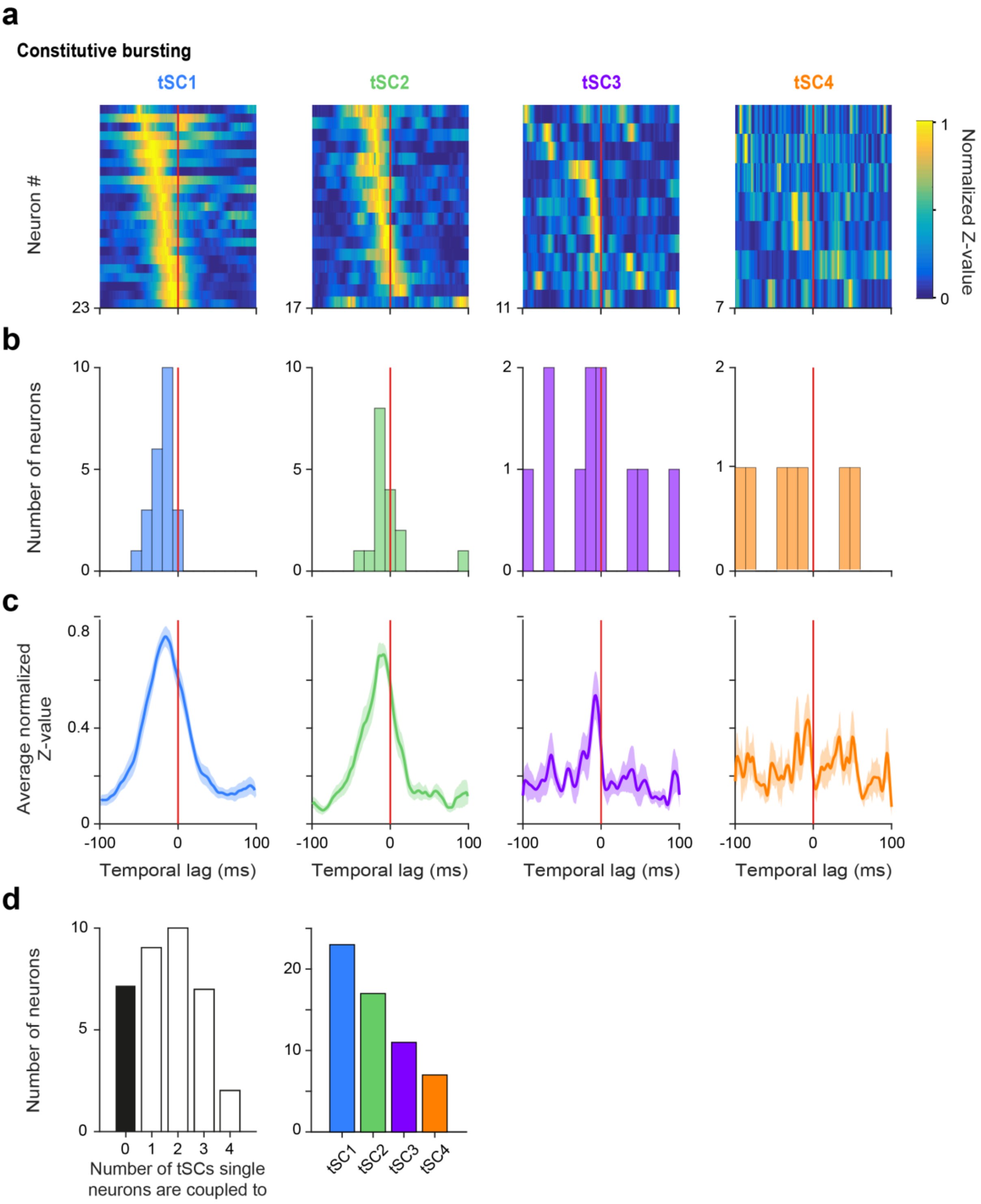

Supplementary Figure 9. Constitutive bursting MS neurons' firing predicts tSCs signals.

a Normalized Rayleigh's Z-value as a function of temporal lag between hippocampal tSCs and MS spike trains. Constitutive bursting MS neurons coupled to the given tSC are shown. Peak Z-values at negative lags indicate that MS signals predict future tSC values. **b** Histograms showing the distribution of time lags across constitutive bursting MS neurons that realize the maximal phase locking as quantified by the Z-values, separately for each tSC. **c** Average normalized Z-value of tSC-coupled constitutive bursting MS neurons as a function of time lag. Error shade represents SEM. **d** Left, histogram of the number of tSCs single constitutive bursting MS neurons are coupled to. Right, number of constitutive bursting MS neurons phase-coupled to each tSC. Source data are provided as a Source Data file.

Supplementary Figure 10

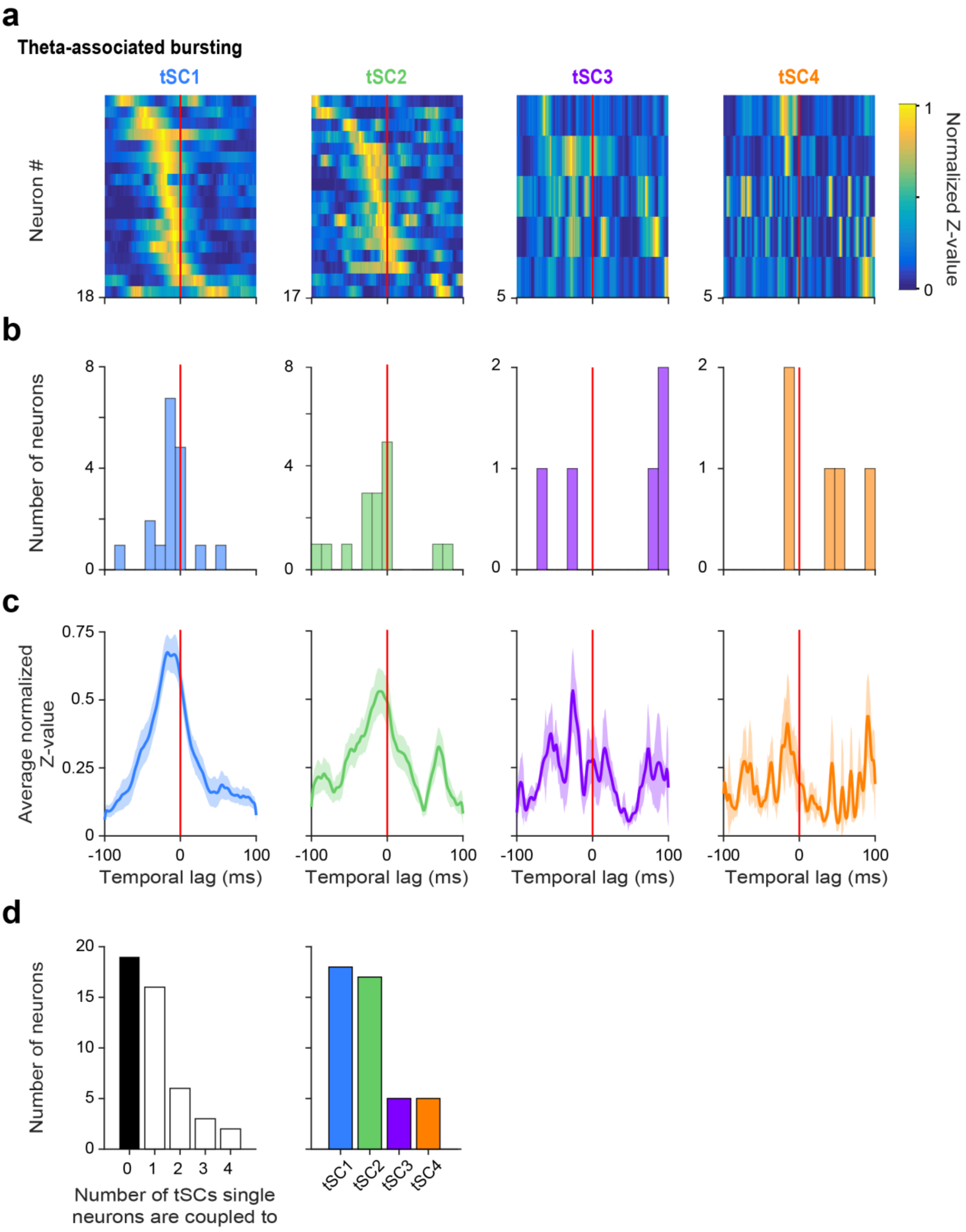

Supplementary Figure 10. Theta-associated bursting MS neurons' firing predicts tSCs signals.

a Normalized Rayleigh's Z-value as a function of temporal lag between hippocampal tSCs and MS spike trains. Theta-associated bursting MS neurons coupled to the given tSC are shown. Peak Z-values at negative lags indicate that MS signals predict future tSC values. **b** Histograms showing the distribution of time lags across theta-associated bursting MS neurons that realize the maximal phase locking as quantified by the Z-values, separately for each tSC. **c** Average normalized Z-value of tSC-coupled theta-associated bursting MS neurons as a function of time lag. Error shade represents SEM. **d** Left, histogram of the number of tSCs single theta-associated bursting MS neurons are coupled to. Right, number of theta-associated bursting MS neurons phase-coupled to each tSC. Source data are provided as a Source Data file.

Supplementary Figure 11

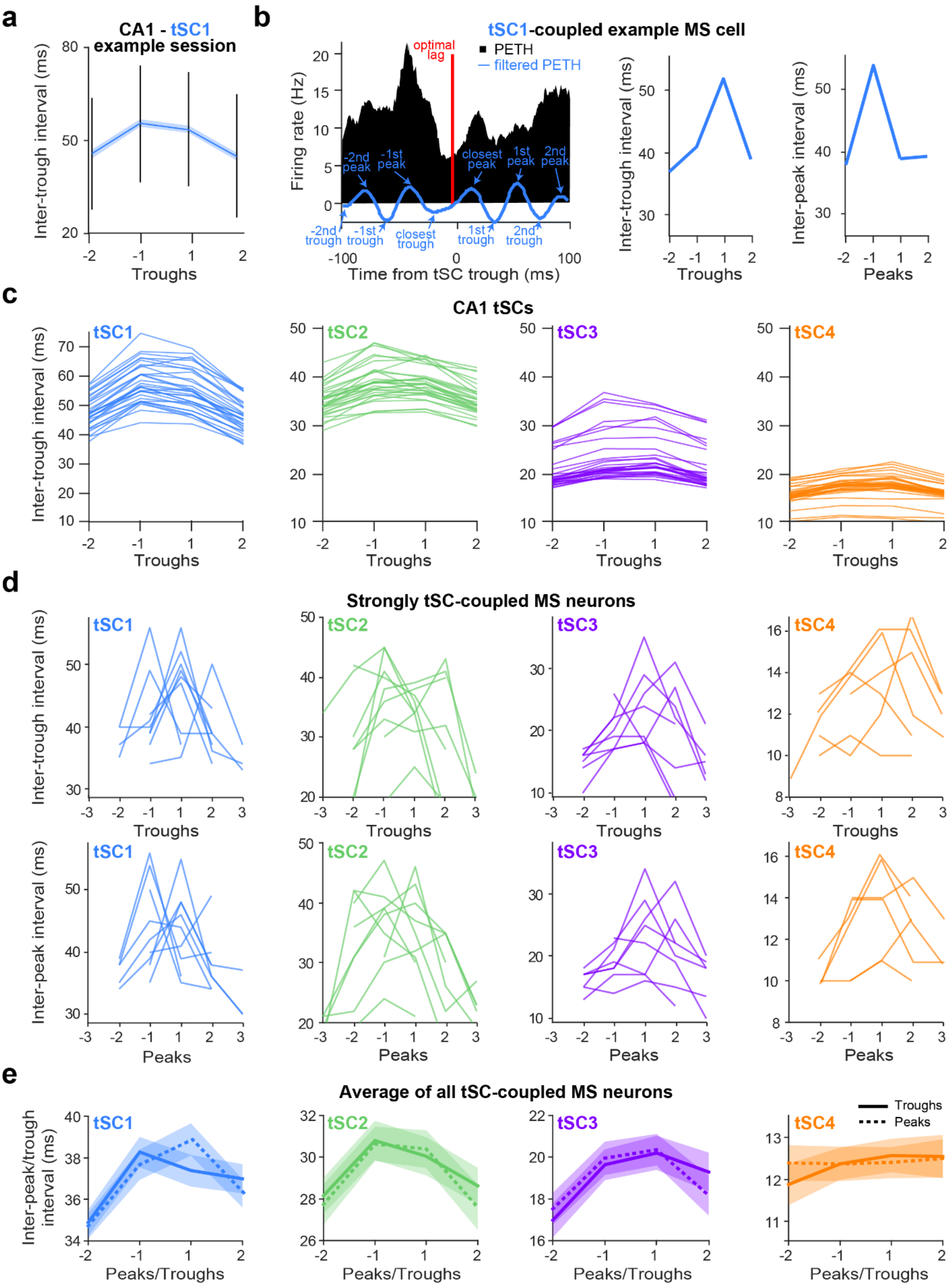

Supplementary Figure 11. Frequency accommodation of CA1 tSCs is reflected in the firing of MS neurons.

a Average tSC1 inter-trough intervals preceding (-2, -1) and following (+1, +2) the largest amplitude trough in an example session. Error bars show the standard deviation to highlight the large variability, while the shaded areas represent the standard error of the mean to demonstrate that the trends revealed are statistically reliable due to the large number of cycles (n = 423) analyzed. **b** Left, peri-event time histogram (PETH) of an example neuron triggered on the largest troughs of CA1 tSC1 cycles from the same session as in panel **a**. The blue line shows the bandpass filtered PETH (18 Hz-35 Hz), which was used to find tSC1-related peaks and troughs before and after the optimal time lag (red line) that realizes the strongest phase locking. Right, inter-trough and inter-peak intervals of the filtered firing rate signal of the example neuron. **c** Average tSC inter-trough intervals for each session (n = 32). **d** Inter-trough and inter-peak intervals of the peaks and troughs of the 10 most tSC-coupled neurons for each tSCs. **e** Average inter-peak/trough intervals of all MS neurons coupled to a given tSC. The shaded areas represent the standard error of the mean. Source data are provided as a Source Data file.

Supplementary Figure 12

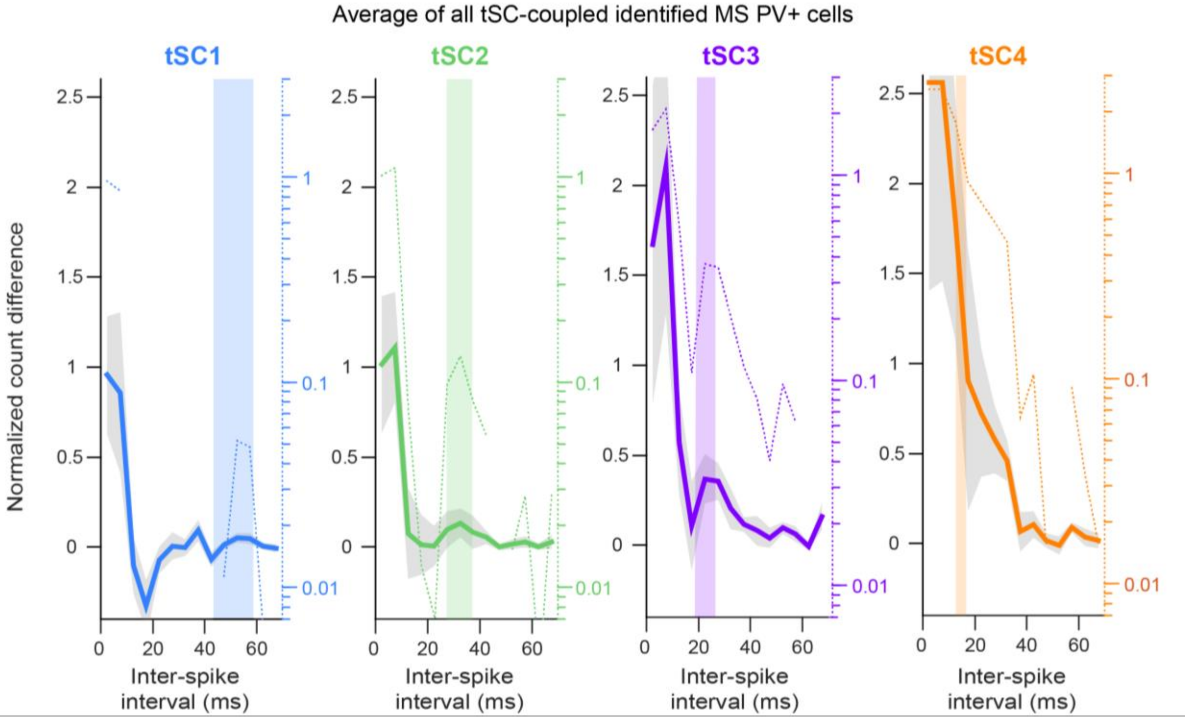

Supplementary Figure 12. Intra-burst frequency of the PV+ MS neuron population is correlated with CA1 tSC presence

Difference between the inter-spike interval histograms during theta cycles with the most and least tSC content (see colored and grey histograms in the examples in Supplementary Figure 7), normalized by firing rate and averaged across identified PV+ tSC-coupled MS neurons. The solid line shows the difference on a linear scale (y-axis on the left), while the dashed line on a logarithmic scale to highlight the difference at slower frequencies (y-axis on the right; note that negative differences cannot be displayed on the logarithmic scale). The error shades show the standard error of the mean. The colored rectangles show the frequency ranges of the tSCs. Source data are provided as a Source Data file.

Supplementary Figure 13

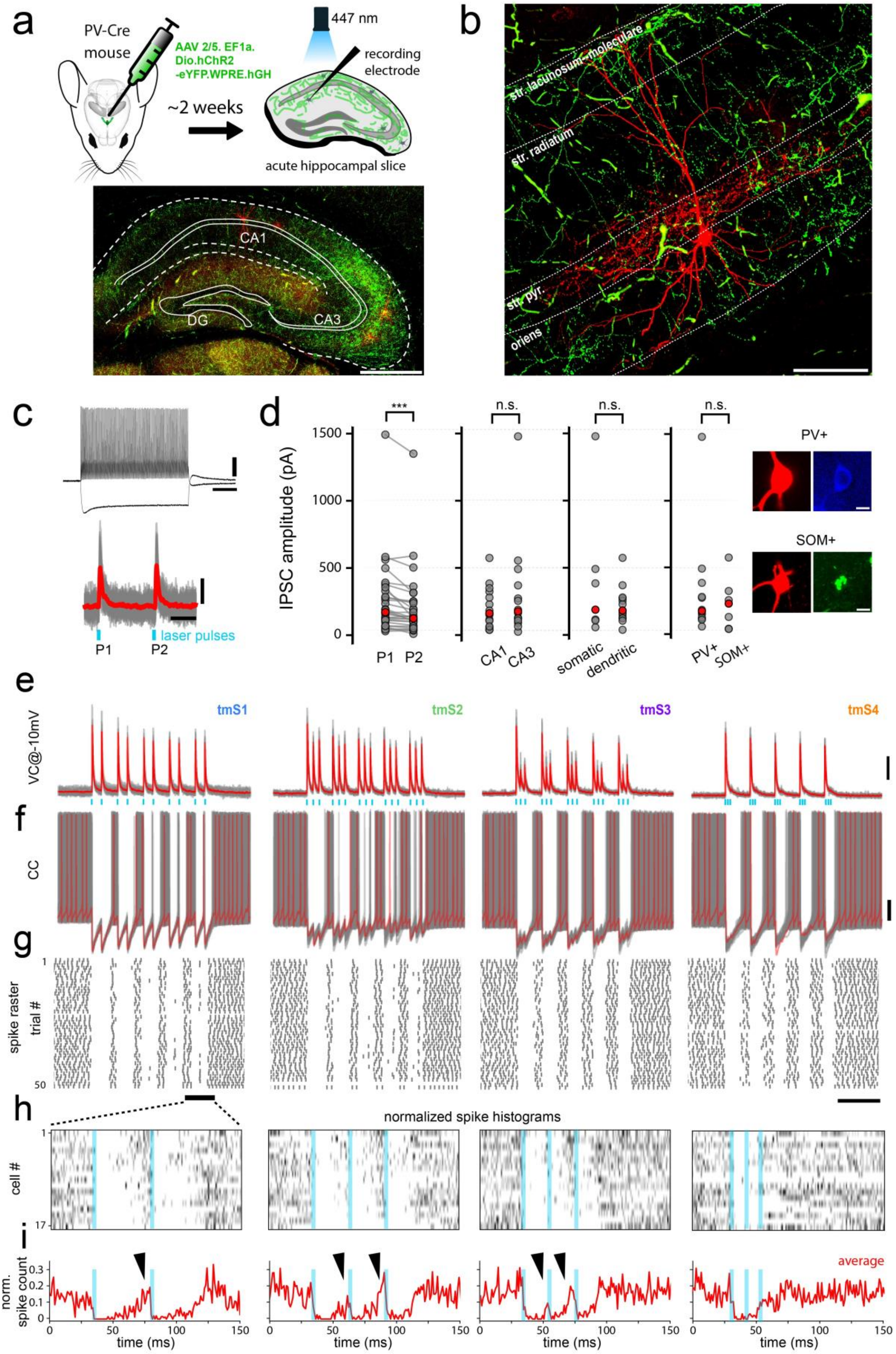

Supplementary Figure 13. CA1 and CA3 PV+ and SOM+ interneurons are targeted by PV+ MS fibers.

a Top, schematic of the acute slice electrophysiology experimental design (Petrucco, Luigi. (2020). Mouse head schema. Zenodo. https://doi.org/10.5281/zenodo.3925903). Cre-dependent AAV vector containing ChR2-eYFP was injected into the MS of PV-Cre animals (n = 6). Coronal acute hippocampal slices were prepared to characterize the PV+ MS inputs to hippocampal interneurons using a series of tmS patterns. Bottom, example of a hippocampal coronal slice with interneurons recorded in the CA3 and CA1 regions (red, biocytin) and PV+ axons arising from the MS expressing ChR2-eYFP (green). Scale bar, 500 µm. **b** Example image of a perisomatic PV+ fast-spiking interneuron recorded in the CA1 region, receiving PV+ MS inhibitory inputs. Scale bar, 100 µm. **c** Somatic current injection evoked spiking pattern (top) and evoked inhibitory currents in response to optogenetic stimulation (2 ms pulse width, 2 pulses @22Hz) of MS fibers (bottom) of the example neuron presented in panel **b** (n = 10 trials overlayed in grey, average in red). Scale bars: top, 200 ms, 20 mV; bottom, 20 ms, 100 pA. **d** Comparison of IPSC amplitudes evoked optogenetically by pulse pairs show moderate but significant short-term depression (n = 29 recorded cells; 2 pulses delivered @22 Hz; P1 and P2 medians, 165.41 and 119.94 pA, respectively, $p = 1.240 \times 10^{-4}$, two-sided Wilcoxon signed-rank test). No significant differences were found between IPSC amplitudes evoked in CA1 versus CA3 (n = 15 and n = 14, respectively; p = 0.621, two-sided Mann-Whitney U-test), soma-targeting versus dendrite-targeting (n = 7 and n = 13; p = 0.817, two-sided Mann-Whitney U-test) or PV+ versus SOM+ (n = 12 and n = 7; p = 0.773, two-sided Mann-Whitney U-test) interneurons. Example image of a PV+ and a SOM+ neuron receiving PV+ MS input are shown on the right (scale bar, 10 µm). **e** Evoked inhibitory currents in response to optogenetic stimulation at different tmS patterns (n = 10 trials overlayed in grey, average in red; scale bar, 100 pA). **f** Effect of tmS stimulation on the spiking of the recorded neuron in panel **e** (n = 50 trials in grey, a single trial is shown in red; scale bar, 20 mV). **g** Raster plot of action potentials of the example neuron for the same tmS patterns (scale bar, 200 ms). **h** Normalized spike histograms of n = 17 cells upon tmS stimulation corresponding to the marked time window in panel **g**. Each line represents normalized spike histogram of a recorded cell, with darker colors indicating higher values. **i** Average normalized spike histograms for the n = 17 cells. Arrowheads highlight peaks in histograms indicating spike modulation by tmS1-3, absent for tmS4. Source data are provided as a Source Data file.

Supplementary Figure 14

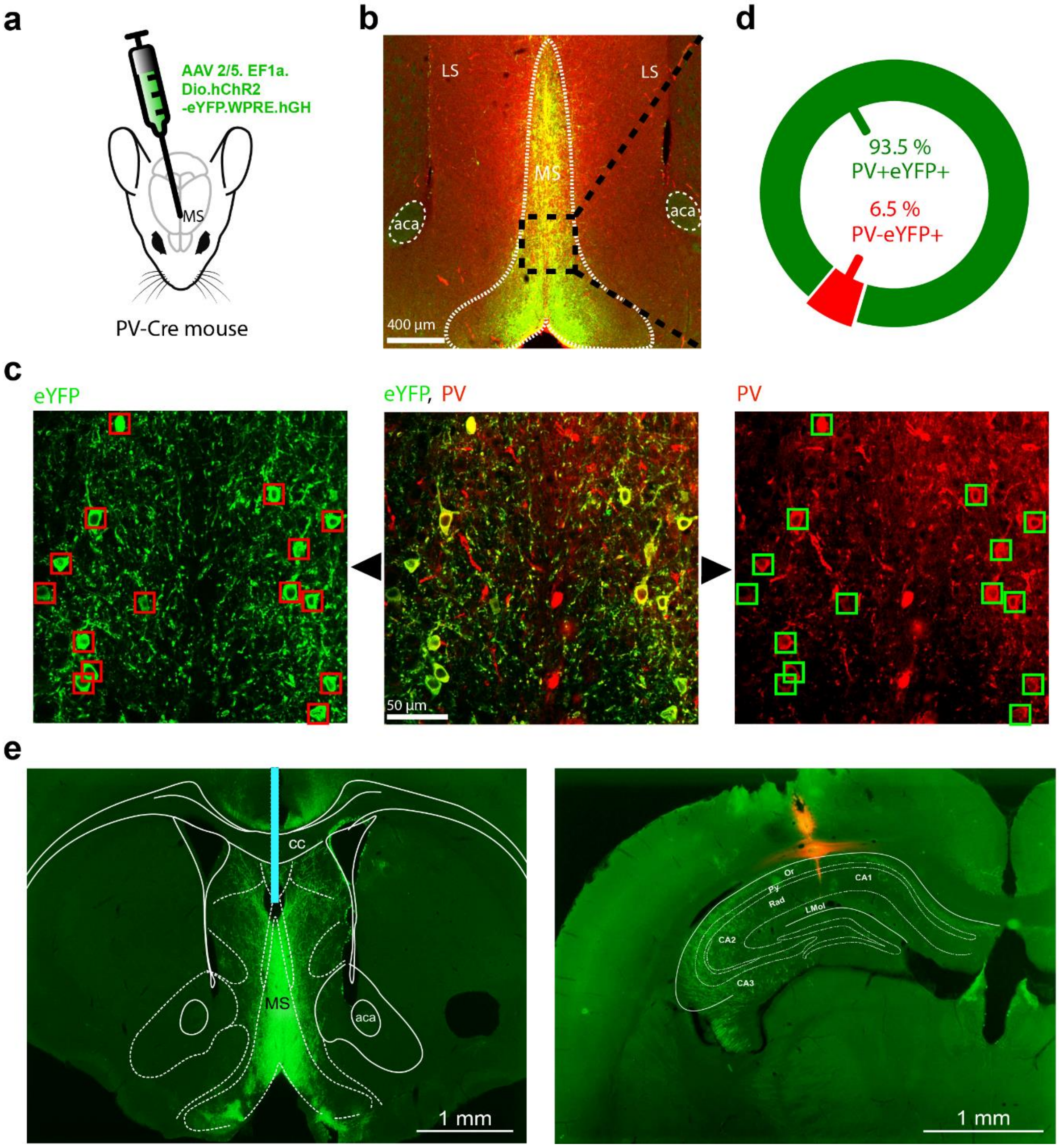

Supplementary Figure 14. Immunohistochemical verification of the cellular identity of the optogenetically stimulated MS neurons targeted in PV-Cre mice and the implant positions

a Schematic of the virus injection experiments (Petrucco, Luigi. (2020). Mouse head schema. Zenodo. https://doi.org/10.5281/zenodo.3925903). **b,c** eYFP and PV expression in the medial septum. The framed area in panel **b** is enlarged in panel **c**. **d** Proportion of PV-expressing neurons among those expressing eYFP (n = 308 cells from n = 3 mice). **e** Fluorescent micrographs of coronal sections showing the position of the optic fiber in the MS (left) and the silicon probe in the CA1 (right) in the same mouse (representative example out of 7 mice;

green, eYFP expressed in septal PV neurons; red, DiI applied on the silicon probes). The blue line indicates the track of the optic fiber above the MS. The corresponding section of the mouse brain atlas[165] was fitted based on anatomical landmarks. aca, anterior commissure anterior part; cc, corpus callosum; Or, oriens layer; Py, stratum pyramidale; Rad, stratum radiatum; LMol, stratum lacunosum moleculare.

Supplementary Figure 15

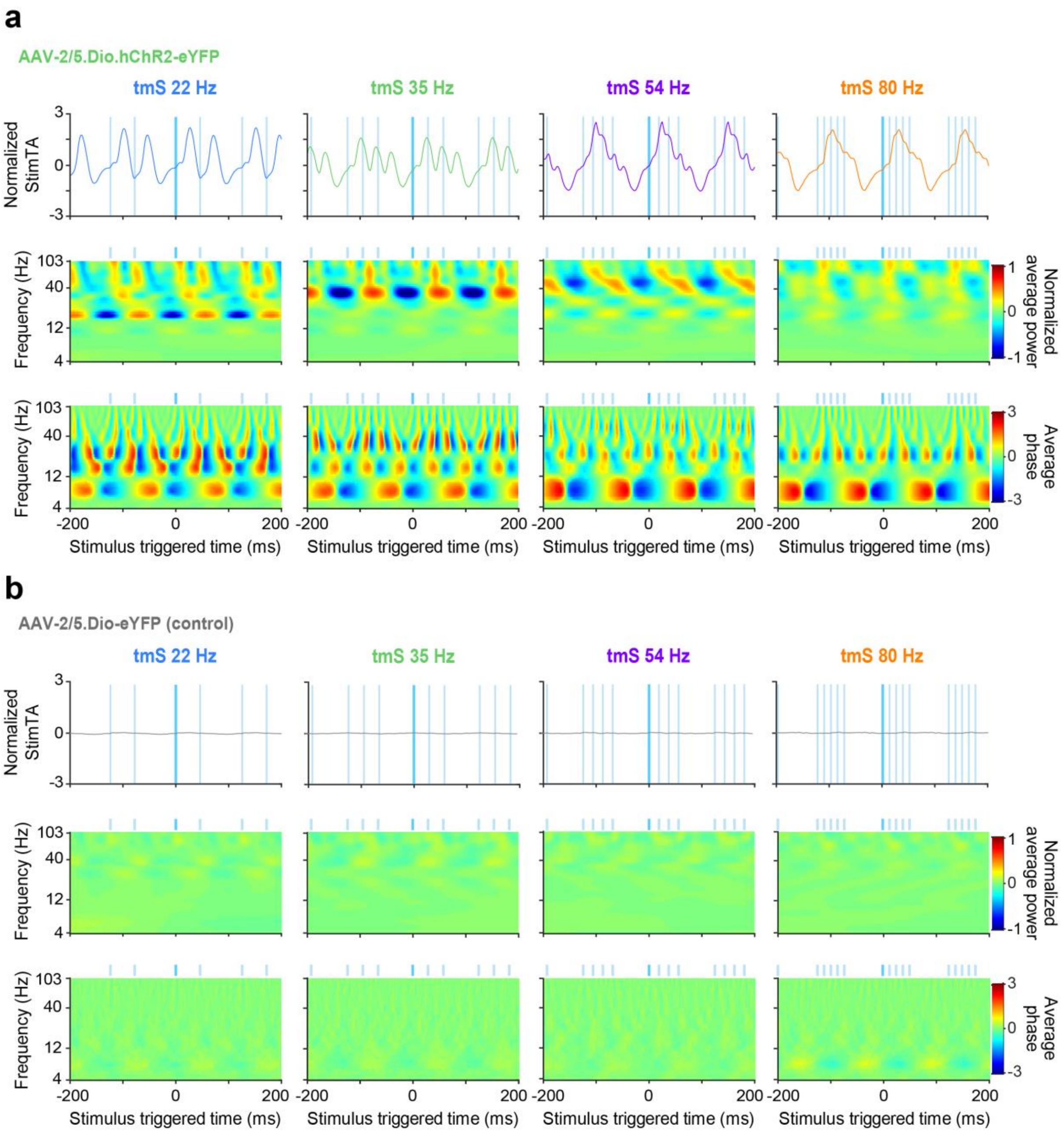

Supplementary Figure 15. Stimulation-evoked tSC-like activity patterns in the CA1

a,b Stimulus triggered averages (StimTA, top) of the raw CA1 LFPs and stimulus triggered spectrograms (middle, power; bottom, phase) for the different tmS protocols applied on PV-expressing MS neurons (**a**) and in the animals injected with the control virus (**b**). Blue lines indicate times of blue laser light pulses.

Supplementary Figure 16

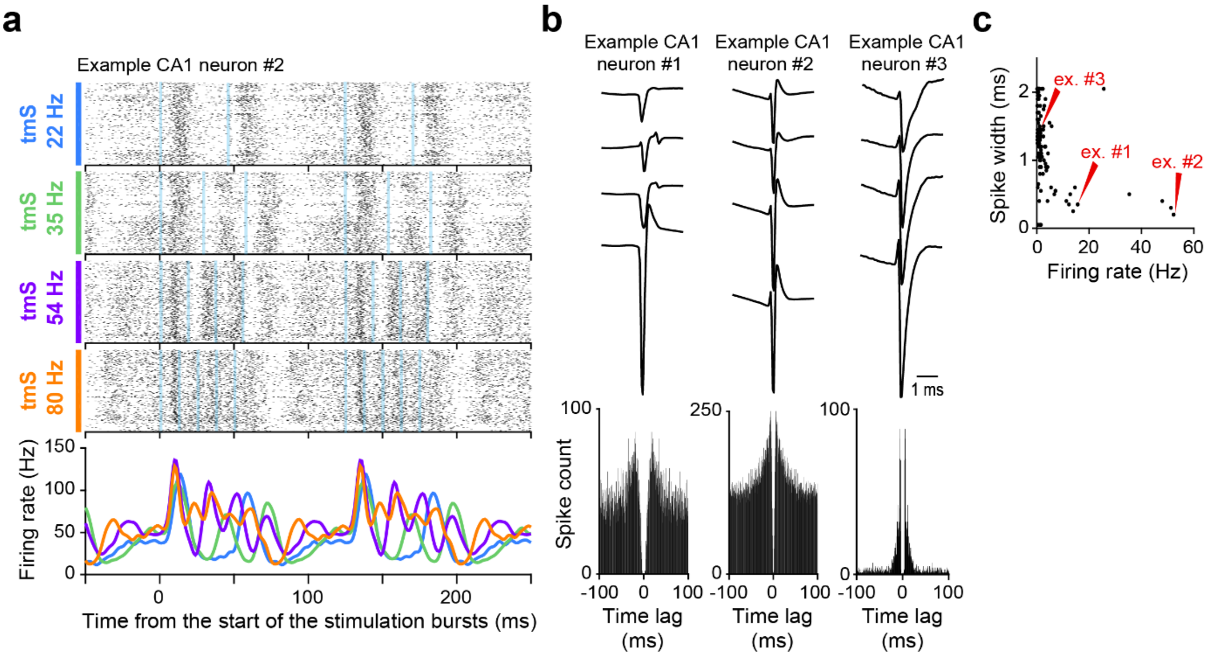

Supplementary Figure 16. Identification of CA1 putative interneurons

a Spike rasters and peri-stimulus time histograms of a putative CA1 interneuron aligned to tmS onset, partitioned by tmS frequency (blue lines, photostimulation). **b** Top, average extracellular action potential shapes for the putative interneuron examples in Fig. 5c (Example CA1 neuron #1) and in Supplementary Figure 11a (Example CA1 neuron #2), compared to a typical putative pyramidal cell (Example CA1 neuron #3). Four channels with the largest spike amplitudes are presented. Bottom, autocorrelograms of the same example neurons. Note the characteristic differences in spike duration and autocorrelograms. **c** Firing rate and spike width (peak-to-valley time) distribution of the recorded CA1 single units from the sessions of the example neurons. Example neurons are marked by red arrowheads. Source data are provided as a Source Data file.

Supplementary Figure 17

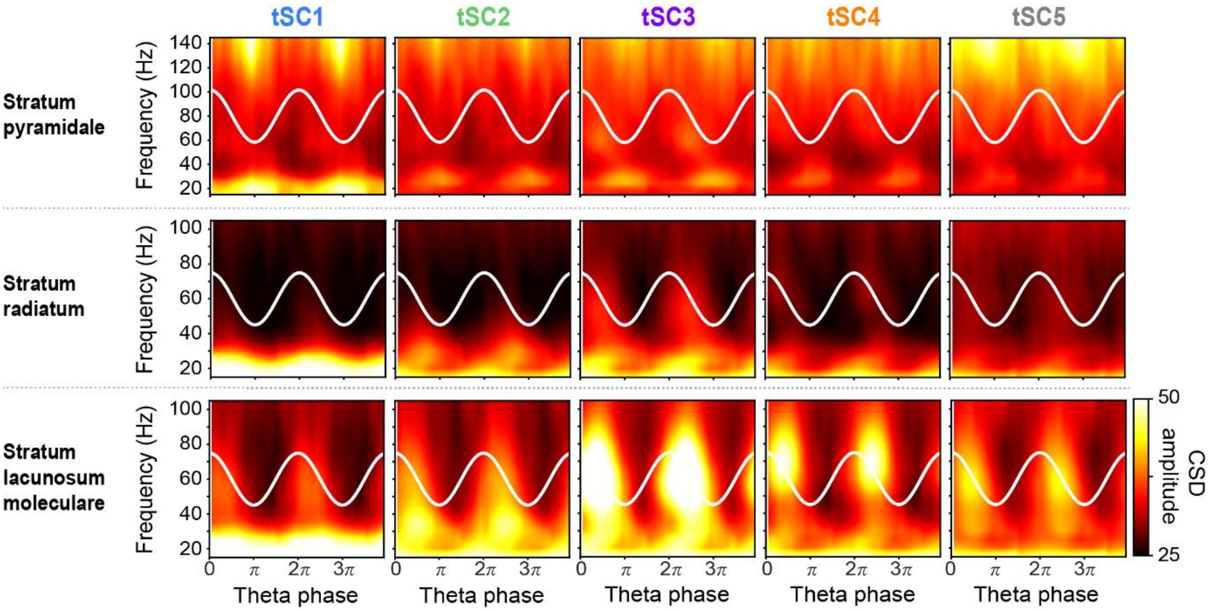

Supplementary Figure 17. Laminar profile of tSC-related currents

Mean amplitude of supra-theta CSD signals as a function of theta phase in different layers of the CA1 from an example session. Theta cycles expressing different tSCs were analyzed separately. Note that the upper limit of the y-axis is extended in the stratum pyramidale panel to visualize fast gamma related currents. Two theta cycles are shown, indicated by the white cosine curves.

Supplementary Figure 18

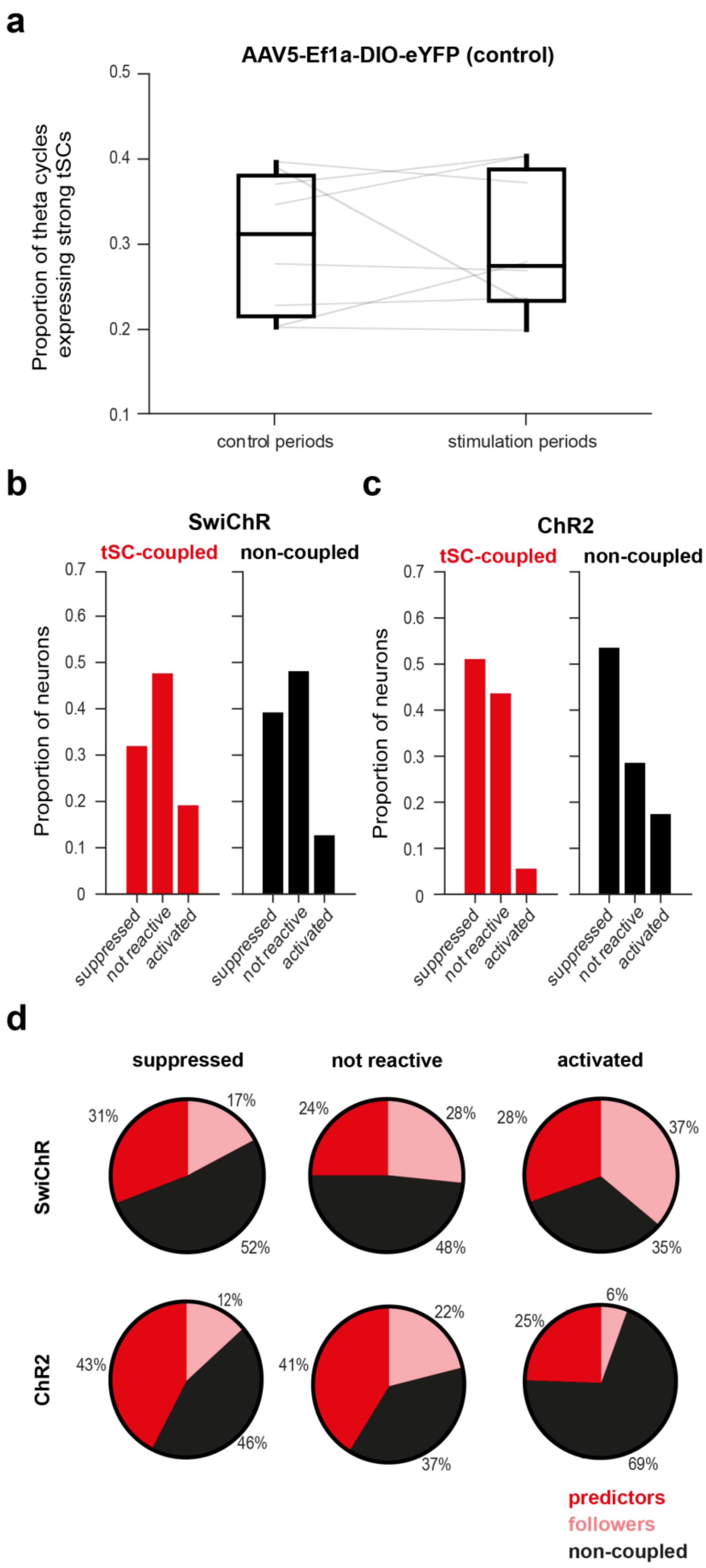

Supplementary Figure 18. The impact of optogenetic manipulations of the hippocampo-septal pathway on MS neurons with different tSC-coupling.

a Proportion of theta cycles expressing tSCs during stimulation and control periods in mice injected with the eYFP control virus. Gray lines connect data points belonging to the same recording sessions (n = 8). Boxes and whiskers show median, interquartile range and non-outlier range. Two-sided Wilcoxon signed-rank test, p = 0.7422. **b** Proportion of MS neurons that showed more than 10% firing rate increase or decrease upon SwiChR-mediated inhibition of the hippocampo-septal projections, shown separately for tSC-coupled and non-coupled MS neuron populations. **c** Same as in panel **a** during optogenetic activation of the hippocampo-septal projections. **d** Proportion of tSC-coupled 'predictor' neurons (maximal tSC phase-locking strength realized by negative time lags), tSC-coupled 'follower' neurons (maximal tSC phase-locking strength realized by positive time lags) and not tSC-coupled neurons among the activated, not reactive and suppressed groups of MS neurons. Source data are provided as a Source Data file.

Supplementary Figure 19

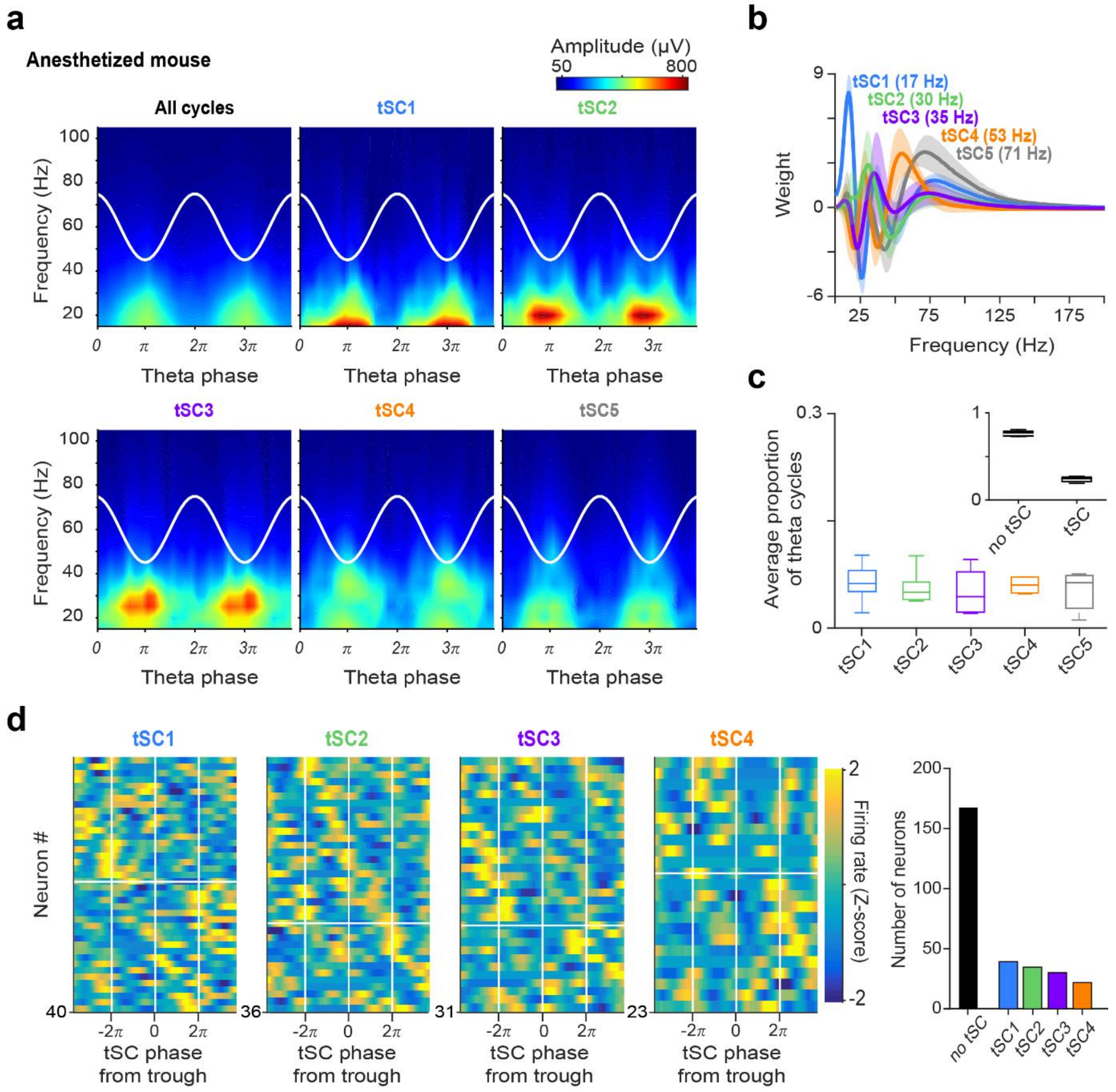

Supplementary Figure 19. MS neurons show phase coupling to hippocampal tSCs in anesthetized mice

a Mean amplitude of supra-theta spectral components as a function of theta phase was computed from the raw LFP of an example session of an anesthetized mouse, for all cycles (top left) and for cycles strongly expressing a given tSC. Two theta cycles are shown, indicated by the white cosine curves. **b** Average tSCs spectra in anesthetized mice (n = 5). Error shades show the standard error of the mean. Peak frequencies are shown in the brackets. **c** Proportion of theta cycles expressing each tSC (n = 5 sessions). Insets show the proportion of theta cycle expressing any of the tSCs. Boxes and whiskers show median, interquartile range and non-outlier range. **d** Left, Z-scored phase histograms of all tSC-coupled MS neurons, sorted into

four groups based on the tSC they are coupled to (blue, low firing rate; yellow, high firing rate). Zero phase corresponds to tSC troughs (white vertical lines). Cells within each group were sorted by their preferred phase in two blocks: top, cells with maximum firing rate before the most negative tSC trough; bottom, cells with maximal firing after the most negative tSC trough. Right, number of neurons phase-coupled to a given tSC. Source data are provided as a Source Data file.

Supplementary Figure 20

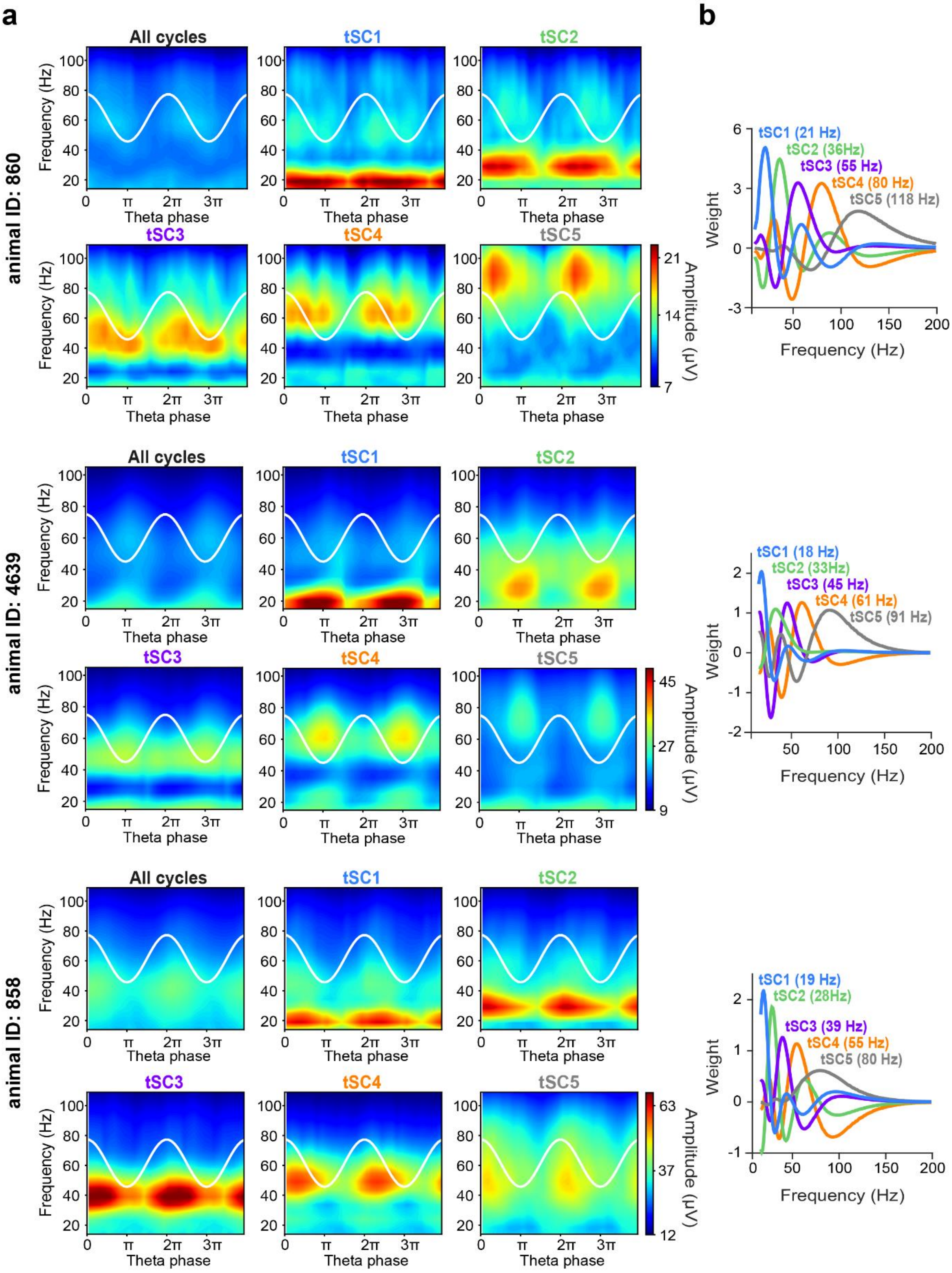

Supplementary Figure 20. Theta nested spectral components in the ventral hippocampal LFP of mice in an elevated plus maze.

a Mean amplitude of supra-theta spectral components as a function of theta phase in three example mice, for all cycles (top left) and for cycles strongly expressing a given tSC. Two theta cycles are shown, indicated by white cosine curves. **b** The frequency content of each tSC in the same example sessions as in panel **a**. Peak frequencies are shown in brackets. Data downloaded from https://datadryad.org/stash/dataset/doi:10.7272/Q6ZP44B9[84].
