# Supplementary material for: The medial septum controls hippocampal supra-theta oscillations
Source: Nat Commun. 2023 Oct 10;14:6159. doi: 10.1038/s41467-023-41746-0 (PMC10564782; doi:10.1038/s41467-023-41746-0)
Supplement: Supplementary file 3 — Reporting Summary [file 41467_2023_41746_MOESM3_ESM.pdf]

nature portfolio

Corresponding author(s): Dr. Balázs Hangya

Last updated by author(s): Aug 2, 2023

Reporting Summary

Nature Portfolio wishes to improve the reproducibility of the work that we publish. This form provides structure for consistency and transparency in reporting. For further information on Nature Portfolio policies, see our Editorial Policies and the Editorial Policy Checklist.

Statistics

For all statistical analyses, confirm that the following items are present in the figure legend, table legend, main text, or Methods section.

n/a	Confirmed	
☐	☒	The exact sample size (*n*) for each experimental group/condition, given as a discrete number and unit of measurement
☒	☐	A statement on whether measurements were taken from distinct samples or whether the same sample was measured repeatedly
☐	☒	The statistical test(s) used AND whether they are one- or two-sided<br>*Only common tests should be described solely by name; describe more complex techniques in the Methods section.*
☒	☐	A description of all covariates tested
☐	☒	A description of any assumptions or corrections, such as tests of normality and adjustment for multiple comparisons
☐	☒	A full description of the statistical parameters including central tendency (e.g. means) or other basic estimates (e.g. regression coefficient) AND variation (e.g. standard deviation) or associated estimates of uncertainty (e.g. confidence intervals)
☐	☒	For null hypothesis testing, the test statistic (e.g. *F*, *t*, *r*) with confidence intervals, effect sizes, degrees of freedom and *P* value noted<br>*Give P values as exact values whenever suitable.*
☒	☐	For Bayesian analysis, information on the choice of priors and Markov chain Monte Carlo settings
☒	☐	For hierarchical and complex designs, identification of the appropriate level for tests and full reporting of outcomes
☐	☒	Estimates of effect sizes (e.g. Cohen's *d*, Pearson's *r*), indicating how they were calculated

Our web collection on statistics for biologists contains articles on many of the points above.

Software and code

Policy information about availability of computer code

Data collection	The Open Ephys 0.4.4.1 and the Amplirec 1.1.03 data acquisition systems were used to collect in vivo electrophysiology data and the pClamp11 software was used for in vitro electrophysiology recordings. The Spike2 software was used to apply different optogenetic stimulation bursts.
Data analysis	All MATLAB codes developed in MatlabR2016a to analyze the data are available at https://github.com/kiralyb/MS_mod_tSC. tSCs were extracted with open source tSC extraction python package (https://data.mrc.ox.ac.uk/data-set/tsc).

For manuscripts utilizing custom algorithms or software that are central to the research but not yet described in published literature, software must be made available to editors and reviewers. We strongly encourage code deposition in a community repository (e.g. GitHub). See the Nature Portfolio guidelines for submitting code & software for further information.

Data

Policy information about availability of data

All manuscripts must include a data availability statement. This statement should provide the following information, where applicable:

- Accession codes, unique identifiers, or web links for publicly available datasets
- A description of any restrictions on data availability
- For clinical datasets or third party data, please ensure that the statement adheres to our policy

The awake and anesthetized rodent electrophysiology recording data used in this study have been deposited at https://doi.org/10.6084/m9.figshare.23798184. The

optogenetic stimulation and electrophysiology recording data generated in this study have been deposited at https://doi.org/10.5281/zenodo.8191988. The juxtacellular recording data from anatomically identified neurons used in this study have been deposited at https://doi.org/10.5281/zenodo.8187903. Ventral hippocampal LFP recordings from mice in an elevated plus maze used in this study are available at https://datadryad.org/stash/dataset/doi:10.7272/Q6ZP44B984. All data points underlying means, line graphs, box plots and scatter plots presented in the figures are provided in a Source Data Excel file with further labeled .mat files for panels presenting multidimensional data. Further data is available from the lead contact upon request.

Human research participants

Policy information about studies involving human research participants and Sex and Gender in Research.

Reporting on sex and gender	No human research participants were involved in this study.
Population characteristics	No human research participants were involved in this study.
Recruitment	No human research participants were involved in this study.
Ethics oversight	No human research participants were involved in this study.

Note that full information on the approval of the study protocol must also be provided in the manuscript.

Field-specific reporting

Please select the one below that is the best fit for your research. If you are not sure, read the appropriate sections before making your selection.

☒ Life sciences ☐ Behavioural & social sciences ☐ Ecological, evolutionary & environmental sciences

For a reference copy of the document with all sections, see nature.com/documents/nr-reporting-summary-flat.pdf

Life sciences study design

All studies must disclose on these points even when the disclosure is negative.

Sample size	Sample size was chosen based on other studies in the field, most importantly Petersen & Buzsáki 2020, Lasztóczi & Klausberger 2016, Belluscio et al. 2012, Lopes-dos-Santos et al. 2018, Kocsis et al. 2022, Salib et al. 2019.
Data exclusions	The recordings from 6 anesthetized mice, 1 anesthetized rat and 6 juxtacellularly labeled neuron were excluded from the phase-coupling analysis due to the lack of sufficient hippocampal theta-gamma activity.
Replication	Each experiment was repeated under the same conditions with the same analysis in 4-7 animals with consistent results. The analyzes of the previously published juxtacellularly labeled neurons recorded from head-fixed mice (Figure 5) showed consistent results with the extracellularly recorded neurons from awake freely moving mice (Figures 3 and 4). Despite differences in the gamma oscillations in awake and anesthetized animals, a smaller proportion of MS neurons exhibited similar phase coupling to hippocampal theta-nested spectral components in anesthetized animals, strengthening the argument that the MS has a general role in hippocampal oscillation genesis.
Randomization	For the optogenetic stimulation experiment, animals were randomly allocated into the test and the EYFP control group. Other experiments in this study did not involve separate experimental groups.
Blinding	The optogenetic stimulation experiment was not blinded to group allocation, since the stimulation protocol and the analyzes was automated. Blinding is not applicable to the other experiments, as animals were not allocated to groups.

Reporting for specific materials, systems and methods

We require information from authors about some types of materials, experimental systems and methods used in many studies. Here, indicate whether each material, system or method listed is relevant to your study. If you are not sure if a list item applies to your research, read the appropriate section before selecting a response.

Materials & experimental systems

n/a	Involved in the study
	☒ Antibodies
☒	Eukaryotic cell lines
☒	Palaeontology and archaeology
	☒ Animals and other organisms
☒	Clinical data
☒	Dual use research of concern

Methods

n/a	Involved in the study
☒	ChIP-seq
☒	Flow cytometry
☒	MRI-based neuroimaging

Antibodies

Antibodies used

primary antibody against PV (PV 27, Swant, Switzerland, 1:2000); primary antibody against GFP (Thermofisher Scientific, USA, cat#A10262, 1:1000); primary antibody against SOM (Origene, #AP3346SU-N, 1:200); Alexa 488 conjugated goat anti-chicken (Thermofisher Scientific, USA, Cat#A11039, 1:1000), Alexa 594 conjugated donkey anti-rabbit (Jackson ImmunoResearch Europe Ltd., UK, cat#711585152, 1:500); Alexa 647 conjugated donkey anti-rabbit (Jackson ImmunoResearch Europe Ltd., UK, cat#711605152, 1:500)

Validation

- Chicken anti-GFP antibody. From the manufacturers datasheet: 'No staining in mice not injected with eGFPexpressing virus'. Thermo Fisher Scientific Cat# A10262, RRID: AB_2534023
- Rabbit anti-somatostatin antibody, Origene, cat#AP3346SU-N ('Specificity: Recognizes Somatostatin-14 '); PMID: 37205047
- Primary antibody against PV. From the manufacturers product description (https://www.swant.com/pdfs/x_Rabbit_anti_parvalbumin_PV27.pdf): 'Antiserum PV27 labels a subpopulation of neurons in the normal brain with high efficiency, but does not stain the brain of parvalbumin knock out mice.'

Animals and other research organisms

Policy information about studies involving animals; ARRIVE guidelines recommended for reporting animal research, and Sex and Gender in Research

Laboratory animals

We used adult SOM-IRES-Cre mice (n = 10; all males; weight: 28-30 g, 3-5 months old) for the chronic freely moving experiments, adult wild type mice (n = 11; 7 males; 6 mice excluded due to less than 360 detected theta cycles; 22-30 g, 3-5 months old) for acute mouse recordings, adult wild type Wistar rats (n = 7; all males; one rat excluded due to less than 360 theta cycles; 200-400 g, 2-5 months old) for acute rat recordings and adult PV-IRES-Cre mice (n = 19; 6 males; 22-30 g, 2-4 months old) for slice electrophysiology, acute awake optogenetic stimulation experiments and verification of the ChR2 expression in PV-expressing neurons.

Wild animals

This study did not involve wild animals.

Reporting on sex

Hippocampal oscillations were examined in both sexes under awake conditions and we did not observe sex-related differences in the theta-gamma phase-amplitude coupling. Chronic electrophysiology recordings were carried out in male mice, because males tolerate chronic implants better due to weight considerations. Nevertheless, optogenetic stimulation of parvalbumin-expressing MS neurons evoked hippocampal theta-nested oscillations in female mice in line with our findings from chronic recordings performed in male mice, thus suggesting that the MS has a similar role in hippocampal oscillation genesis in both sexes. Due to these study design complexities and sample size considerations, we refrained from post-hoc sex-based analyses.

Field-collected samples

This study did not involve field-collected samples.

Ethics oversight

All experiments were approved by the Animal Care and Use Committee of the Institute of Experimental Medicine or the Animal Care Committee of the Research Centre for Natural Sciences and the Committee for Scientific Ethics of Animal Research of the National Food Chain Safety Office and were performed according to the 2010/63/EU Directive of the EC Council.

Note that full information on the approval of the study protocol must also be provided in the manuscript.
